# Microbial Diversity in Hummock and Hollow Soils of Three Wetlands on the Qinghai-Tibetan Plateau Revealed by 16S rRNA Pyrosequencing

**DOI:** 10.1371/journal.pone.0103115

**Published:** 2014-07-31

**Authors:** Yongcui Deng, Xiaoyong Cui, Marcela Hernández, Marc G. Dumont

**Affiliations:** 1 Key Laboratory of Virtual Geographic Environment of the Ministry of Education, Nanjing Normal University, Nanjing, China; 2 University of the Chinese Academy of Sciences, Beijing, China; 3 Department of Biogeochemistry, Max Planck Institute for Terrestrial Microbiology, Marburg, Germany; Argonne National Laboratory, United States of America

## Abstract

The wetlands of the Qinghai-Tibetan Plateau are believed to play an important role in global nutrient cycling, but the composition and diversity of microorganisms in this ecosystem are poorly characterized. An understanding of the effects of geography and microtopography on microbial populations will provide clues to the underlying mechanisms that structure microbial communities. In this study, we used pyrosequencing-based analysis of 16S rRNA gene sequences to assess and compare the composition of soil microbial communities present in hummock and hollow soils from three wetlands (Dangxiong, Hongyuan and Maduo) on the Qinghai-Tibetan Plateau, the world’s highest plateau. A total of 36 bacterial phyla were detected. Proteobacteria (34.5% average relative abundance), Actinobacteria (17.3%) and Bacteroidetes (11%) had the highest relative abundances across all sites. Chloroflexi, Acidobacteria, Verrucomicrobia, Firmicutes, and Planctomycetes were also relatively abundant (1–10%). In addition, archaeal sequences belonging to Euryarchaea, Crenarchaea and Thaumarchaea were detected. Alphaproteobacteria sequences, especially of the order Rhodospirillales, were significantly more abundant in Maduo than Hongyuan and Dangxiong wetlands. Compared with Hongyuan soils, Dangxiong and Maduo had significantly higher relative abundances of Gammaproteobacteria sequences (mainly order Xanthomonadales). Hongyuan wetland had a relatively high abundance of methanogens (mainly genera *Methanobacterium*, *Methanosarcina* and *Methanosaeta*) and methanotrophs (mainly *Methylocystis*) compared with the other two wetlands. Principal coordinate analysis (PCoA) indicated that the microbial community structure differed between locations and microtopographies and canonical correspondence analysis indicated an association between microbial community structure and soil properties or geography. These insights into the microbial community structure and the main controlling factors in wetlands of the Qinghai-Tibetan Plateau provide a valuable background for further studies on biogeochemical processes in this distinct ecosystem.

## Introduction

Soil microbial communities in wetland systems play an important role in biogeochemical cycles and are crucial to the functions of wetland systems [1]. In recent years, owing to the advantages of next-generation sequencing, research on microbial diversity in various natural wetlands has rapidly developed. For example, the 16S rRNA tag-encoded pyrosequencing approach has been adopted to study microbial diversity in different natural wetlands, such as a *Sphagnum*-dominated northern wetland of Russia [2], marshes in eastern Massachusetts [3], peatlands in the Glacial Lake Agassiz region [4], and freshwater wetlands in northern Virginia [5]. Although considerable progress has been achieved in characterizing the microbial diversity in natural wetlands, further research is still needed.

The Qinghai-Tibetan Plateau (av. 4000 m a.s.l.) is the largest and highest plateau on Earth. The area of natural wetlands on the Qinghai-Tibetan Plateau is estimated at 13.3×l0^4^ km^2^. In contrast to boreal wetlands located at high latitude regions, wetlands in this region are at low latitude and high altitude. Additionally, they have distinguishing features of hummock-hollow microtopography and Sedge-dominated vegetation [6]. Wetlands on the Qinghai-Tibetan Plateau are very sensitive to climate change and they play a vital role in terrestrial carbon storage [7]. Also, these wetlands provide many important ecological services, such as ensuring the productivity of rangelands and providing grazing ground for thousands of livestock animals. Despite the importance of these wetlands, the composition of soil microbial communities in this distinct ecosystem has not been fully investigated. The microbiological research in this region has been primarily restricted to specific functional groups of microorganisms, such as methanogens [8], [9] and methanotrophs [10], [11], or microbes involved in other specialized processes [12], [13]. In addition, a few cultivation-independent studies using denaturing gradient gel electrophoresis (DGGE) fingerprinting and limited clone library construction have been carried-out to assess the bacterial diversity in wetlands of this region [14].

Due to practical limitations of the techniques previously employed, limited phylogenetic resolution and coverage of the microbial diversity in wetland environments on the Qinghai-Tibetan Plateau is available. Furthermore, many important aspects are still not investigated. First, despite numerous wetlands unevenly distributed on the Qinghai-Tibetan Plateau [15], the differences in microbial communities between these wetlands at different geographical locations are mostly unexplored. Second, no study has focused on variations in the microbial community structures between hummock and hollow soils, which is the common microtopography in wetlands on this plateau [6]. Lastly, recent studies using molecular techniques have provided evidence that environmental factors, particularly soil properties, influence the bacterial community structure in several terrestrial and aquatic systems including wetland environments [16]–[19]; however, this has not been investigated in wetlands of the Qinghai-Tibetan Plateau.

The aim of the present study was to detect the composition and diversity of the bacterial and archaeal community present in hummock and hollow soils of three geographically separate Sedge-dominated wetlands on the Qinghai-Tibetan Plateau. 454-pyrosequencing of 16S rRNA genes was used to analyze the relative abundance of major bacterial and archaeal groups. We also analyzed the correlation of environmental factors to the microbial community structures in this distinct ecosystem.

## Materials and Methods

### Sampling permissions

Permission for sampling at the Hongyuan wetland was obtained from the Administrative Office of the Hongyuan Riganqiao Wetland Natural Reserves. No specific permissions were required for the sampling in Dangxiong and Maduo wetlands. The field studies did not involve endangered or protected species.

### Site description and soil sampling

Three Sedge-dominant wetlands located on the Qinghai-Tibetan Plateau (Figure S1) and characterized by hummock-hollow microtopography were chosen for the study. The Dangxiong (DX) wetland (30°28′11.00N, 91°03′42.02E, 4290 m a.s.l.) is located in DX county of Tibet nearby the southern edge of the Nyainqêntanglha Mountains and in the basin of two tributaries of the Lhasa River. The main vegetation is *Kobresia* and *Carex* in hummocks and hollows, respectively. The average annual air temperature is 1.3°C and the average annual rainfall is 476.8 mm [20]. The second wetland is the Riganqiao/Hongyuan (HY) peatland (33°06′52.73N, 102°38′ 37.39E, 3459 m a.s.l.) located in HY County (northwestern Sichuan Province), and is located in the northeastern edge of the Qinghai-Tibetan Plateau. The climate of HY is a continental high-plateau monsoon climate, with mean annual air temperature of 1.1°C and average annual precipitation of 650 mm [21]. The vegetation cover is primarily *Carex meyeriana* and *Carex muliensis* [22]. Both DX and HY wetlands have mature peat habitats [21], [23]. The third wetland is Maduo (MD) wetland (34°38′10.30N, 98°01′46.29E, 4229 m a.s.l.) located in the center of Sanjiang River Source Natural Reserve [7]. Typical peat has not been formed in MD wetland. The average annual air temperature of MD is −4.1°C and the mean annual precipitation is 303.9 mm [24]. The dominant plants in MD wetland are *Kobresia* (in hummocks) and *Carex* (in hollows).

At the time of sampling, the water table was ca. 15 cm above hollow soil surfaces and ca. 5 cm below the top hummock soils. Soil cores from three hummocks and three hollows in these three wetlands were collected from 0–5 cm soil depth in August 2011. Soil samples were kept in a cool box during transportation and were stored in the laboratory at 4°C. Soil pH was determined after transportation of samples to a laboratory using a compound electrode and a soil-to-water ratio of 1∶2.5. Soil moisture (SM) was measured by drying soils at 105°C for 24 h. Soil organic carbon (SOC) and total N (TN) were determined by dichromate oxidation and Kjeldahl digestion, respectively. Available P (AP) in the soil was measured by the sodium bicarbonate extraction-molybdenum-antimony anti-colorimetry method.

Some abbreviations are used in the following paragraphs and figures for simplicity: DXa and DXb represent the hummock soils and the hollow soils of the DX wetland, respectively; HYa and HYb represent the hummock soils and the hollow soils of the HY wetland and MDa and MDb represent the hummock soils and the hollow soils of the MD wetland.

### DNA extraction, real-time PCR and pyrosequencing

Soil DNA was extracted from 0.4 g fresh soil (18 samples, 3 hummock soils and 3 hollow soils from 3 wetlands) using the NucleoSpin soil kit (Macherey-Nagel, Düren, Germany). DNA quality was verified using a NanoDrop 1000 spectrophotometer (Thermo Fisher Scientific, Schwerte, Germany) and diluted 100-fold in water for quantitative real-time PCR (qPCR) analysis. The assays targeting bacterial 16S rRNA and archaeal 16S rRNA were performed using the Taqman real-time PCR System as described previously [25]. The assays were performed using an iCycler instrument (Bio-Rad) and the associated software. DNA extracts were diluted 10-fold before the PCRs for pyrosequencing. Primers F515 (5′-GTGCCAGCMGCCGCGGTAA) and R806 (5′-GGACTACVSGGGTATCTAAT) were used to amplify 16S rRNA genes. Individual PCRs were barcoded by 6-bp molecular barcodes specific for each sample for the subsequent identification. PCRs were performed in 50-µl volumes containing 5 µl 10×AccuPrime PCR Buffer II (Life Technologies, Darmstadt, Germany), 0.4 mM of each primer, 1 µl of Taq AccuPrime (Life Technologies), 1 µl of template and 39 µl sterile water. Cycling was performed with an initial denaturation at 94°C for 5 min followed by 27 cycles: 50°C 30 s, 68°C 30 s, 94°C 30 s, and a final extension at 68°C for 10 min on an Eppendorf Mastercycler instrument (Eppendorf, Hamburg, Germany). PCR products from each tagged primer set were pooled and purified using the GenElute PCR Clean-Up Kit (Sigma, Taufkirchen, Germany), and DNA concentrations were determined using a Qubit instrument (Life Technologies). Finally, samples were pooled in an equimolar concentration for 454-pyrosequencing. Pyrosequencing was carried-out at the Max Planck-Genome-Centre Cologne and performed using standard procedures using a Roche 454 Genome Sequencer GS FLX+ instrument. The 454 pyrosequencing reads (raw data) were deposited under the study accession number SRP033622 in the NCBI Sequence Read Archive.

### Post-run sequence analysis

The taxonomic assignment of pyrosequencing reads and preprocessing of sequences was performed using USEARCH (v. 7.0.1090) [26], Qiime (v. 1.7.0) [27] and Mothur (v. 1.27) [28] software platforms, as described below. All raw sequences were first analyzed and the OTU table was created using the UPARSE pipeline [26] (http://www.drive5.com/usearch/manual/uparse_cmds.html). In this pipeline, sequences were first sorted based on barcodes and sequences were removed from further analysis when they were shorter than 200 bp, contained ambiguous bases or homopolymers greater than 6 bp in length. Chimeras were removed using UCHIME [29]. Operational taxonomic units (OTUs) were picked from the good sequences with a cutoff value of 97% sequence identity. Taxonomic classification was carried-out in Qiime with the Bayesian classifier and the SILVA reference database with a confidence threshold of 80%. Rarefaction curves and diversity indices including microbial community diversity (Shannon index), richness (Chao1) and coverage were calculated in Mothur.

A heatmap representation of the relative abundance of OTUs between samples was constructed using R (http://www.r-project.org/). A Hellinger transformation of the OTU counts was performed using the decostand function in the vegan package [30]. Principal components analysis (PCA) was performed using prcomp and the result indicated that PC1, PC2 and PC3 explained 25%, 15% and 12% of the variance, respectively. In order to select the OTUs explaining most of the differences between samples, the 25 OTUs with highest loadings of PC1, 15 OTUs of PC2 and 12 OTUs of PC3 were chosen to construct the heatmap [31]. A total of 42 unique OTUs were obtained since some of the OTUs were selected from more than one PC. The OTU abundances were converted to percentage of reads from each sample and Manhattan distances were calculated and the heatmap constructed using the heatmap.2 function in gplots [32]. The taxonomy of the selected OTUs was added separately and ANOVA was performed using SPSS Statistics (20.0) Software (IBM).

UniFrac is a β-diversity measure that uses phylogenetic information to compare environmental samples. Unweighted UniFrac distances [33] in principle coordinate analysis (PCoA) were used to compare the microbial community composition among samples. Datasets were subsampled to 4,130 sequences. Representative OTU sequences from UPARSE were aligned in Mothur, and then a newick formatted phylogenetic tree was built using FastTree in Qiime. Lastly, a 3D PcoA plot was created in Qiime and visualized by KiNG (v. 2.16).

Two-way analysis of variance (ANOVA) on microbial diversity indices and relative sequence abundances of major taxa and selected OTUs, and the subsequent statistical analysis of simple main effects and the post hoc pairwise test were all performed by SPSS statistics (20.0) software. To identify environmental factors influencing microbial diversity, canonical correspondence analysis (CCA) was performed between the OTU profiles (subsampled to 4,130 sequences per sample) and environmental factors using the vegan package in R [30].

## Results

### Microbial diversity indices

**Figure 1:**
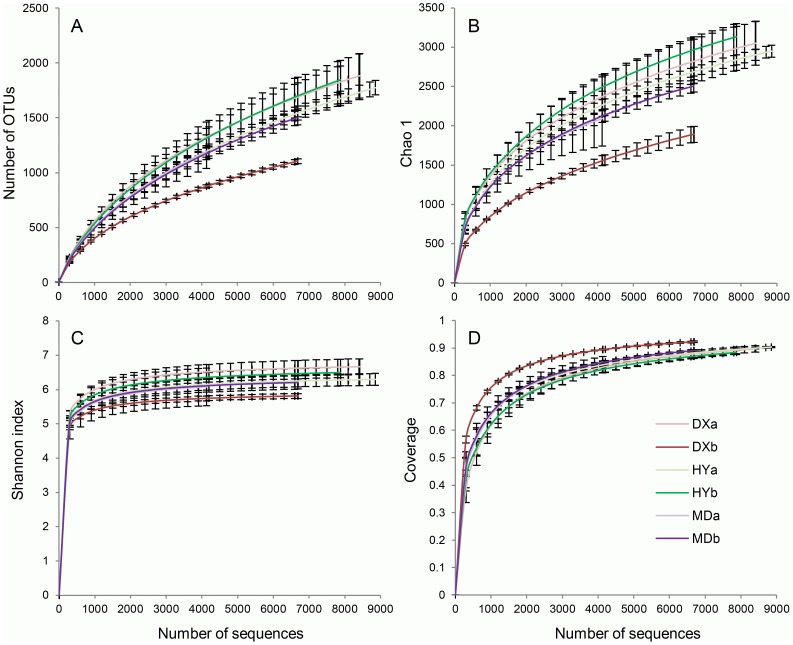
Rarefaction curves showing the relationship between the increase of bacterial α-diversity and the number of randomly sampled sequences. A, number of OTUs observed experimentally; B, diversity predicted by Shannon index; C, richness predicted by Chao1; D, the sample coverage.

A total of 158,939 microbial sequences belonging to 9,907 OTUs were obtained after the quality filtering. A similarity level of 97% was used to identify OTUs and to estimate diversity. At this genetic distance, an average of 1,179 OTUs and Chao1 value of 2,156 were found in the samples at a sequencing depth of 4,130 (Table S1 in File S1). The rarefaction curves of OTUs and Chao1 were not saturated (Figure 1A and B). In contrast, rarefaction curves of Shannon indices approached a plateau at a sequencing depth of 2,000 (Figure 1C). The Shannon diversity indices at a sequencing depth of 4,130 and its rarefaction curves showed that it ranged from 5 to 7 (Figure 1C and Table S1 in File S1). The coverage of all samples at a sequencing depth of 4,130 is above 80% (Figure 1D and Table S1 in File S1).

Two-way ANOVA was used to analyze the effects of locations and microtopographies on all diversity indices, and significant interaction effects were detected (Table S1 in File S1, P<0.05). The simple main effects analysis showed that the main reason for these interaction effects was the low diversity and richness of DXb samples. OTUs, Chao1, Shannon and Coverage indices between hummock and hollow soils were only significantly different in the DX wetland (results not shown).

### The abundance of archaeal taxa

The abundances of Archaea in these three wetlands were significantly different. HY wetland had the highest abundance of Archaea at about 10^8^ 16S rRNA gene copies g^−1^ soil, whereas it was 10^6^–10^8^ copies g^−1^ soil in DX and only 10^6^–10^7^ copies g^−1^ soil in MD wetland (Table 1). The hollow soils in DX and MD wetlands had significantly more archaeal sequences compared with the hummocks.

**Table 1:** Overview of environmental data in hummock and hollow soils of the three wetlands (mean±SD, n = 3).

Sample	TN	SOC	AP	pH	Soil moisture	Gene copy numbers (×10^7^) (g^−1^ fresh soil)	
	g kg^−1^	g kg^−1^	mg kg^−1^		%	16S rRNA Bacteria	16S rRNA Archaea
DXa	7.64±2.28	133.79±7.22	5.13±3.02	6.14±0.12	65.7±0.6	776±58	0.817±0.774
DXb	4.90±0.97	72.81±21.93	5.12±2.48	4.38±0.07	66.9±6.7	1842±409	13.8±2.7
HYa	13.94±1.85	390.36±13.75	4.12±0.24	6.23±0.58	83.5±0.8	1000±62.6	27.4±8.03
HYb	20.56±1.42	331.4±13.79	3.76±0.06	5.73±0.35	82.0±2.7	660±228	25.7±7.45
MDa	3.16±0.3	37.8±6.63	0.56±0.11	8.36±0.06	49.3±3.7	343±189	0.673±0.178
MDb	2.05±0.24	21.95±2.17	0.03±0.03	7.92±0.1	36.3±3.2	706±286	4.65±0.553

The pyrosequencing PCR primers used for the amplification of 16S rRNA genes have specificity for both bacterial and archaeal sequences. A total of 65 archaeal 16S rRNA OTUs from 2,549 sequences were obtained, corresponding to 2.61%, 4.74%, 0.14%, 0.96%, 0.07% and 0.49% of microbial sequences from HYa, HYb, DXa, DXb, MDa and MDb, respectively. These ratios were similar to the ratio calculated using the qPCR data (Table 1), which were 2.74%, 3.89%, 0.11%, 0.75%, 0.2% and 0.66%.

**Figure 2:**
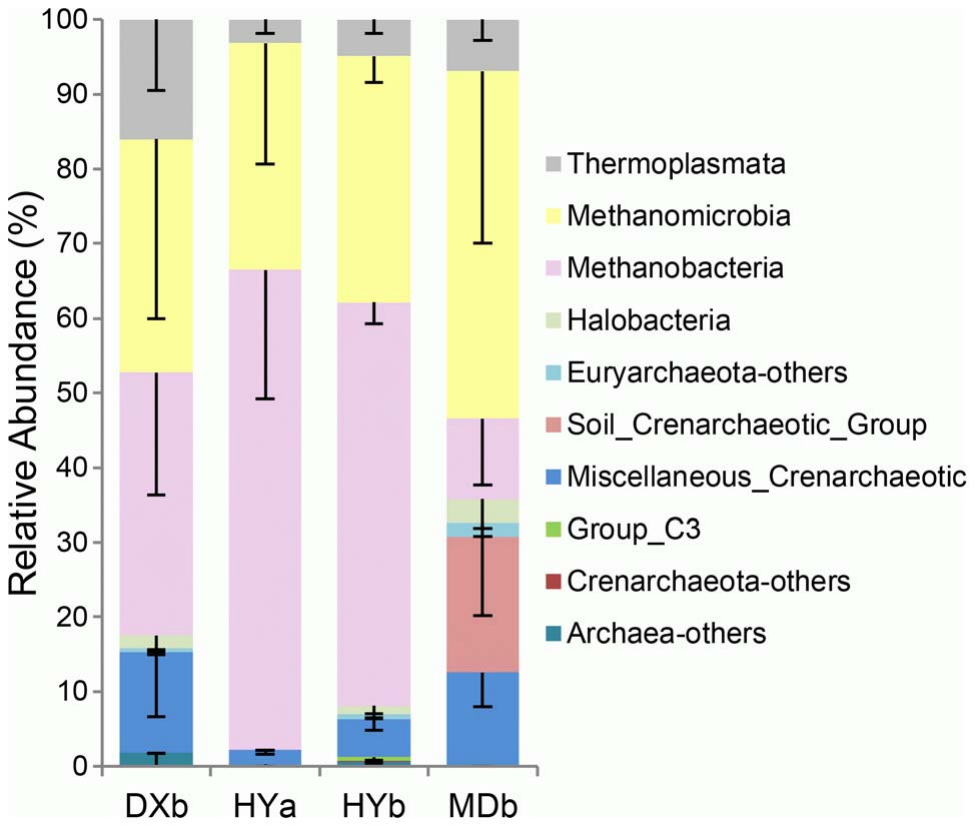
Taxonomic assignment of sequences affiliated with Archaea from samples in different wetlands and microtopographies. Error bars indicate the standard deviation of relative sequence abundance between three replicate samples.

Most samples of DXa and MDa had less than 10 sequences, so these were not included in the taxonomy analysis because the coverage was too low. The archaeal communities were composed of both Crenarchaeota and Euryarchaeota phyla (Figure 2). Crenarchaeota-affiliated sequences were relatively abundant in MDb (30.7%). The crenarchaeotal sequences were mainly affiliated with Miscellaneous Crenarchaeotic Group and Soil Crenarchaeotic Group. Euryarchaeota sequences were dominant in most of the samples and accounted for 97.8%, 93.7%, 84.7% and 69.3% of archaeal sequences from HYa, HYb, DXb and MDb, respectively.

### Relative abundance of various bacterial taxa

**Figure 3:**
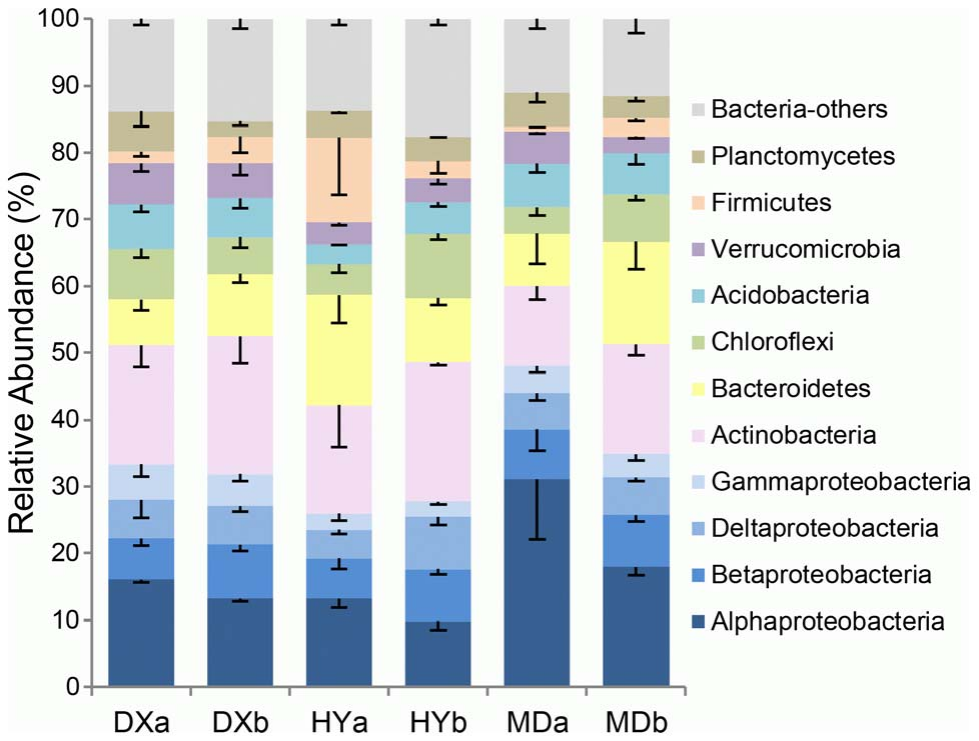
Relative sequence abundances of bacterial phyla and proteobacterial classes. Major taxa detected with average relative sequence abundances >1% are displayed. Column “Bacteria-others” indicates combined relative sequence abundances of all the rare phyla, candidate divisions and of the taxonomically unclassified sequences; rare phyla were defined as having average relative sequence abundances between all samples of <1%.

The 16S rRNA gene sequences retrieved from all samples were affiliated with 36 bacterial phyla and corresponded to 9,772 bacterial species-level OTUs. The relative abundance (>1%) of various phyla and proteobacterial classes is shown in Figure 3. The predominant phylum was Proteobacteria (average 34.5%), including Alphaproteobacteria (16.9%), Betaproteobacteria (7.2%), Deltaproteobacteria (5.8%) and Gammaproteobacteria (3.7%) classes. The community structures were significantly affected by location (P<0.001, Table S2 in File S1). MD soils had a significantly (P<0.001) higher relative abundance of Alphaproteobacteria sequences than DX and HY soils (Figure 3). Also, significantly higher relative abundances of Alphaproteobacteria were obtained from hummocks than hollows (P = 0.004, Figure 3). Orders Rhizobiales and Rhodospirillales comprised 78.6% of sequences in the class Alphaproteobacteria, and Rhodospirillales in particular had higher relative abundance in MD soils compared with the other two wetlands (Figure S2A). The most abundant phylotype within the Betaproteobacteria belonged to the Burkholderiales and the phylotype with the second highest relative abundance could not be classified at a higher level and was named “Others” in Figure S2B. Within Deltaproteobacteria, Myxococcales and Desulfuromonadales were the two orders with highest relative abundance (Figure S2C). Gammaproteobacteria sequences, especially Xanthomonadales, the most abundant order in this class, had significantly lower relative abundances in HY soils compared with the other two wetlands (Figure S2D).

The other phyla with high relative abundances, as shown in Figure 3, were Actinobacteria (17.3%), Bacteroidetes (11%), Chloroflexi (6.4%), Acidobacteria (5.5%), Verrucomicrobia (4.3%), Firmicutes (4.1%), and Planctomycetes (4%). Their relative abundances were significantly different between locations (P<0.05) except for Chloroflexi and Planctomycetes (Table S2 in File S1); however, the relative abundance of Planctomycetes was significantly higher in hummocks than hollows across all samples (P = 0.004, Figure 3) and Chloroflexi was significantly higher in hollows than hummocks in HY and MD samples (P = 0.005, Figure 3). In addition to the above abundant phyla, rare bacterial phyla were also found and are shown in Figure S3. We defined rare phyla as those having an average abundance between all samples of ≤1%. Although Candidate_division_OP8 and Cyanobacteria were rare when considering all samples together, their relative abundances were >1% in HYb and DXb respectively. In total, these rare phyla together constituted a higher proportion of the sequences in hollows compared with hummocks (Figure S3).

Acidobacteria, Bacteroidetes, Chloroflexi and Verrucomicrobia phyla were relatively abundant (>27%) in the study sites. Within the Acidobacteria phylum, Acidobacteria and Holophagae were the two most abundant classes. The relative abundance of the Acidobacteria class was affected by location (P = 0.001, Table S3 in File S1) and had low abundance in HY soils (Figure S4A). In the phylum Bacteroidetes, the most abundant classes were Sphingobacteria and Bacteroidia, which together amounted to 86.6% of the Bacteroidetes phylum (Figure S4B). Sphingobacteria had higher relative abundance in hollow soils compared with hummock soils (P = 0.004, Table S3 in File S1, Figure S4B). The two most abundant classes in the phylum Chloroflexi were Anaerolineae and KD4-96, contributing 75.1% to this phylum (Figure S4C). Anaerolineae was also associated with location (P = 0.001) and had greater relative abundance in hollow soils compared with hummock soils (P<0.001, Table S3 in File S1, Figure S4C). Within the Verrucomicrobia phylum, the classes Spartobacteria and OPB35 had the highest relative abundance (Figure S4D). Spartobacteria was significantly more abundant in hummocks than hollows across all sites (P<0.001, Table S3 in File S1, Figure S4D).

### Methane cycle-related microorganisms

Methanogens and methanotrophs were present in the hummocks and hollows of three wetlands. The HY wetland had the highest abundance of both methanogens and methanotrophs (Table 2). The main classes of methanogens were Methanobacteria and Methanomicrobia, which together accounted for 94.6%, 87.1%, 66.4% and 57.4% of all archaeal reads in HYa, HYb, DXb and MDb, respectively (Figure 2). *Methanobacterium* was the most abundant genus of methanogen in HY wetlands. *Methanosaeta* were only present in DX and HY wetlands, and tended to have higher relative abundance in hollow soils of the HY wetland. *Methanosarcina* had higher relative abundances in hollows of the three wetlands, and also in hummocks of HY wetland. Methanotrophs detected in these wetlands belonged to the phylum Proteobacteria, including type I methanotrophs (Gammaproteobacteria, *Methylobacter*) and type II methanotrophs (Alphaproteobacteria, *Methylocystis* and *Methylosinus*). *Methylocystis* was dominant and had high relative abundance in HY soils compared with the other two sites.

**Table 2:** Percent relative abundance of 16S rRNA genes in the pyrosequencing datasets associated with genera of methanogens and methanotrophs (mean±SD, n = 3).

Taxon	DXa	DXb	HYa	HYb	MDa	MDb
Methanogens						
* Methanobacterium*	0.05±0.07	0.29±0.18	1.62±0.21	2.56±1.51	0.02±0.01	0.04±0.03
* Methanocella*	0	0.02±0.01	0.02±0.02	0.05±0.04	0	0
Candidatus Methanoregula	0±0.01	0±0.01	0.07±0.08	0.27±0.18	0	0
Rice Cluster II	0	0	0.05±0.04	0.11±0.05	0	0
* Methanosaeta*	0.01±0.01	0.06±0.09	0.06±0.02	0.76±0.37	0	0
* Methanosarcina*	0.01±0.01	0.26±0.23	0.60±0.54	0.25±0.12	0	0.21±0.16
Methanotrophs						
* Methylocystis*	0.19±0.32	0.09±0.06	0.69±0.15	0.62±0.12	0.03±0.03	0.04±0.05
* Methylosinus*	0	0±0.01	0.02±0.01	0±0.01	0	0.01±0.02
* Methylobacter*	0	0±0.01	0.02±0.02	0.07±0.08	0	0.01±0.01
* Methylocaldum*	0	0	0.03±0.04	0.05±0.01	0	0
* Methylomonas*	0.01±0.01	0	0.02±0.01	0.02±0.02	0	0

Only genera with >0.01% relative abundance are shown.

### OTU-level microbial β-diversity analysis

**Figure 4:**
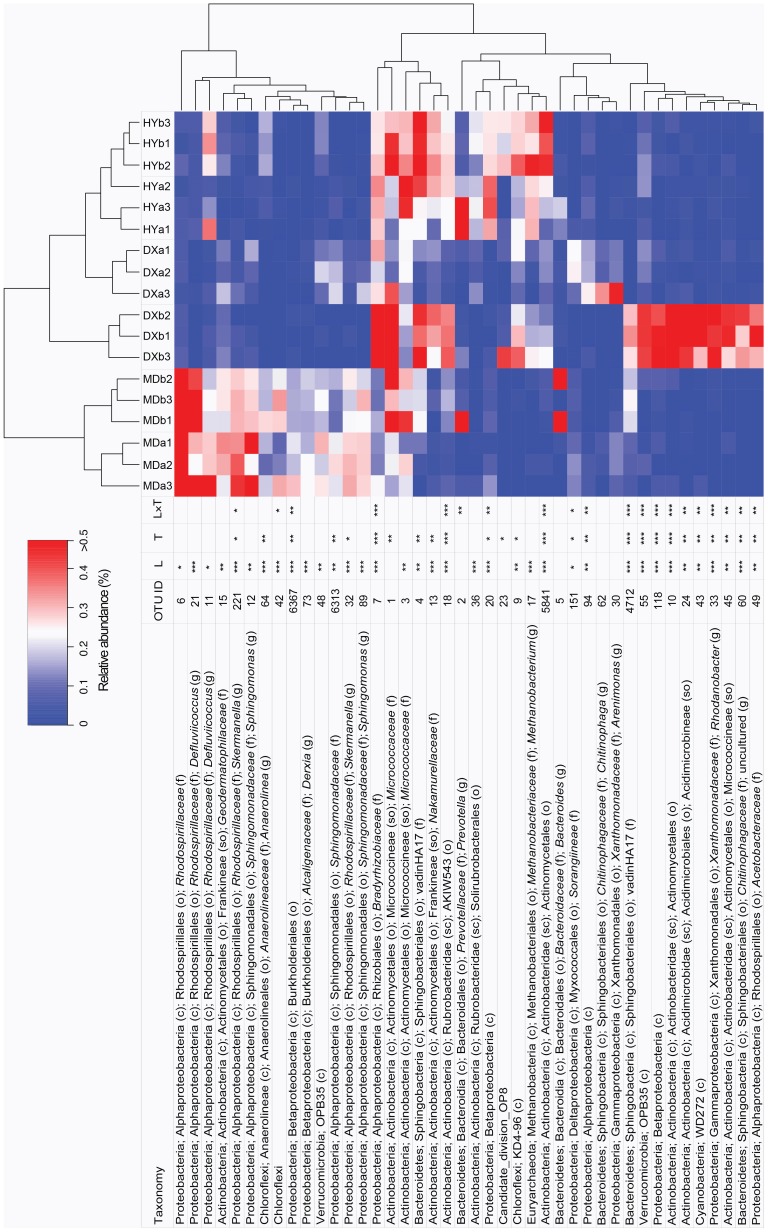
Heatmap showing the relative abundance of selected OTUs. The samples and OTUs are clustered according to Manhattan distances. The colors correspond to the relative abundance of the OTUs in the samples, as indicated by the color legend. The taxonomy of each OTU is provided to the lowest-level attained during the classification. Abbreviations are used to indicate class (c), order (o), suborder (so), family (f) and genus (g). Two-way ANOVA for each OTU was performed using location (L), topography (T) and location and topography (L×T). P-values of the ANOVA are indicated as follows: * (P<0.05), ** (P<0.01), *** (P<0.001).

β-diversity assesses the differences between microbial communities and reflects the dissimilarity between samples. A heatmap was used to intuitively display the differences in OTU relative abundances observed in this study (Figure 4). The OTUs with highest contribution to the PCA ordination were selected. Most of these OTUs (36/42) were significantly different between the three locations based on ANOVA tests (Figure 4). The OTUs fell into five main clusters. The cluster positioned at the top of the heatmap mostly had high relative abundance in the MD wetland only. Among them, eight OTUs belonged to the class Alphaproteobacteria (five OTUs of *Rhodospirillaceae* and three of *Sphingomonadaceae*). The three clusters of OTUs in the middle of the heatmap were mostly associated with high relative abundance in the HY and DX wetlands. Within this group was OTU-17, corresponding to the genus *Methanobacterium*. The cluster at the bottom of the heatmap comprised OTUs that had high relative abundance in the hollows of the DX wetland. The relative abundance of most OTUs (36 of 42) exhibited significant differences based on location, and many (26 of 42) based on topogeography. For example, OTU-7 (belonging to *Bradyrhizobiaceae*), OTU-1 (*Micrococcaceae*) and OTU-4 (Sphingobacteriales, vadinHA17) had higher relative abundance in hollows compared with hummock soils (P<0.01).

**Figure 5:**
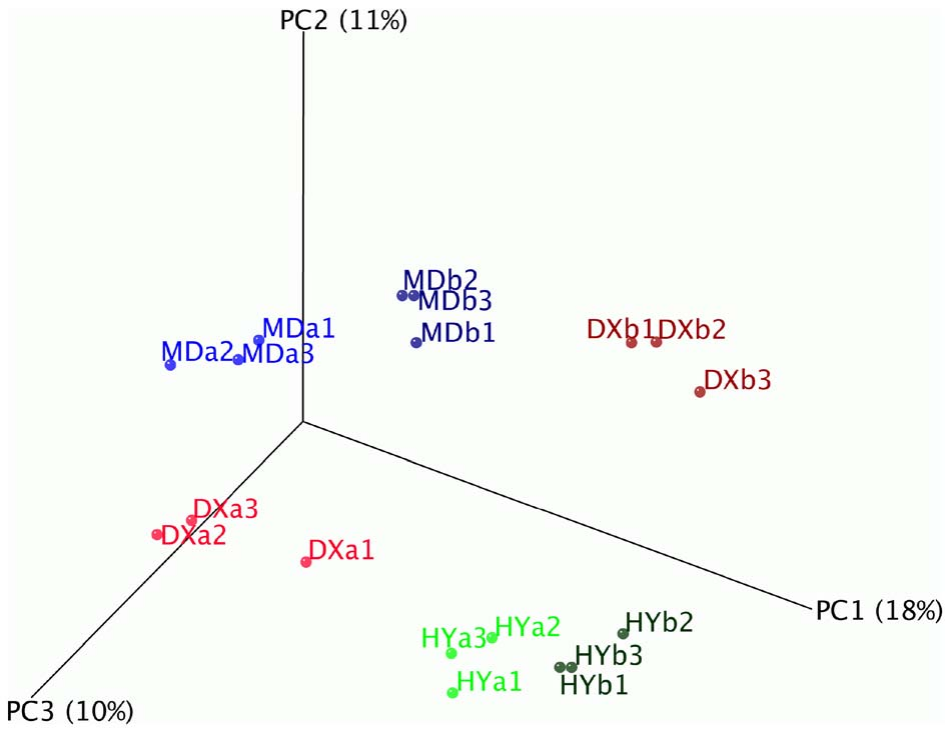
Principal coordinate analysis (PcoA) plot of samples using the unweighted UniFrac distance metric.

A PCoA plot was used to visualize the data based on β-diversity metrics of unweighted UniFrac (Figure 5). There were obvious separate, non-overlapping groups for each of the locations, and hummock samples were also separated from hollow samples. A partial Mantel test was used to estimate the correlation between the OTU level unweighted UniFrac matrix and another matrix derived from either the location or microtopography parameters, while using one of the other location or microtopography as the third “control” distance matrix. The Mantel *r* statistic of 0.55 and P-value of 0.001 indicated a significant relationship between UniFrac distances and wetland locations while controlling for differences in microtopographies. There was also a relatively strong relationship between UniFrac distances and microtopography while controlling for differences in locations (r = 0.41, P = 0.001). These results confirmed that bacterial communities were significantly different between locations and microtopographies.

### Association between microbial community structures and soil variables

**Figure 6:**
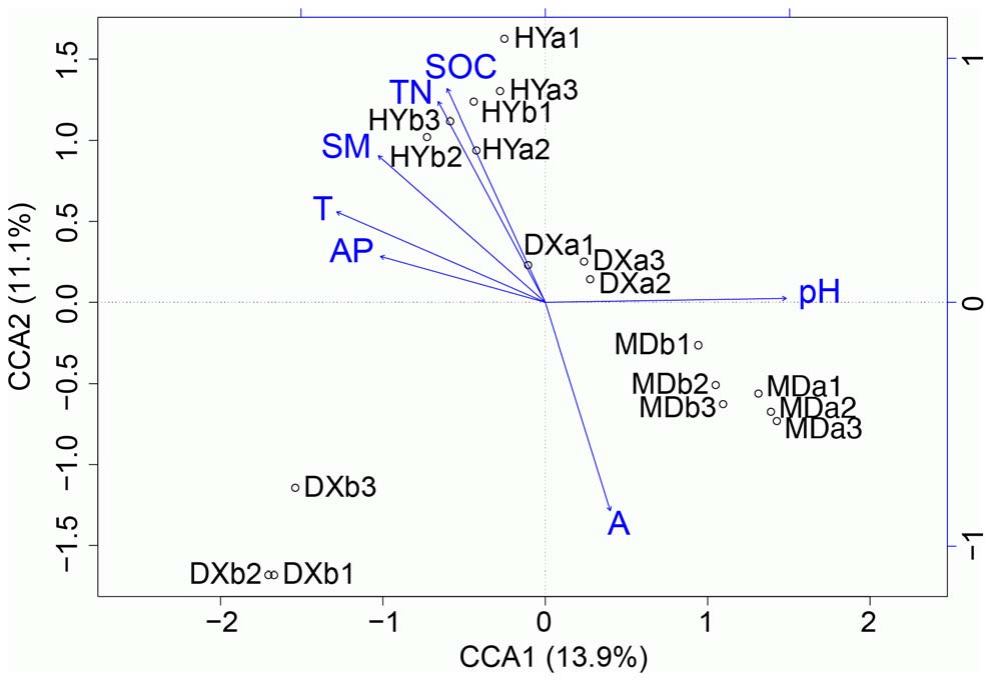
Canonical correspondence analysis (CCA) used to evaluate the effect of soil factors on the bacterial community structure. Soil factors indicated in blue text include AP (available phosphorus), pH, SOC (soil organic carbon), TN (total nitrogen), T (mean annual air temperature), SM (soil moisture) and A (altitude).

The environmental data of the three wetlands are shown in Table 1. We used Mantel’s tests to determine the significance of independent influences of environmental factors (AP, SOC, TN, C:N, pH, SM, plant biomass (PB), mean annual air temperature (T), mean annual precipitation (P) and altitude (A)) on the distribution and diversity of microbial OTUs across different samples. Except plant biomass, significant correlations were found between environmental factors and microbial community structure. The soil factors (AP, SOC, TN, pH and SM) and the location factors (T, P and A) all explained >10% of microbial community variation between samples (Table S4 in File S1). Canonical correspondence analysis (CCA) was used to discern the microbial structure according to environmental parameters. The seven significant environmental variables were selected and included in the CCA plot (Figure 6). pH was the most important contributor to axis 1, which on its own accounted for 13.7% of the variation. These environmental variables together explained 64% of the difference in the microbial communities between the sites.

## Discussion

We analyzed the microbial diversity in different wetlands of the Qinghai-Tibetan Plateau using identical experimental conditions, which is critical for taxon richness estimation. The microbial rarefaction curves of OTUs and Chao1 were not saturated (Figure 1A and B), suggesting we still did not survey the full extent of taxonomic diversity at a taxonomic resolution of 97% sequence identity for the fragments of 16S rRNA genes analyzed. At the sequencing depth of 4,130, the Chao1 and average detected OTUs were lower in hollow soils of DX wetland than other samples; however, even these indices in DXb (Chao1 value of 1,563 and 8,75 OTUs, Table S1 in File S1) were higher than those reported for a northern peatland (Chao1 value of ∼700 and ∼500 OTUs) [2]. The rarefaction curves of the Shannon index approached the plateau phase with more than 2,000 sequences per sample, indicating that Shannon curves were saturated (Figure 1B). The differences in Shannon indices between the three wetlands were not significant; however, relatively lower Shannon indices (6.2) were found in our study site compared with other natural wetlands, e.g. 7.3 in northern peatland of Russia [2] and 6.7 in Yellow River Estuary wetlands [34]. Although we analyzed the sequences with quality controls, some errors may still exist because of PCR error, sequencing error, and the methods employed within the bioinformatic pipeline [35]. Although the Shannon index is relatively insensitive to sequencing and PCR errors [35], it is still sensitive to the bioinformatic processing. Therefore, even with the same high-throughput sequencing method, it is difficult to directly compare the diversity indices obtained using different protocols.

The large bacterial diversity in these wetlands included high relative abundances of Proteobacteria (37.5%), Actinobacteria (17.3%) and Bacteroidetes (11%) (Figure 3). It is not surprising to see the dominance of Proteobacteria and Actinobacteria as they are prevalent in soil of various ecosystems [36]; however, Bacteroidetes had the third highest relative abundance among the bacterial phyla and these were greater than those of wetlands in most other regions. For example, wetlands of Northern Russia [2], a North Carolina coastal plain and the Florida Everglades [19] all had Bacteroidetes totaling less than ∼1%. Bacteroidetes were found to have the highest relative abundance among microorganisms in high-altitude wetlands in Chile [37]. Also, they were found to be the predominant bacteria in Tibetan plateau glaciers [38], and also one of the dominant phyla in sediments [17] and freshwaters [39], [40], [41] of the Tibetan plateau lakes. Therefore, the presence of relatively high abundance of Bacteroidetes in the three wetlands of the Qinghai-Tibetan Plateau may be partly associated with factors shared between these high altitude environments and warrants further investigation into their ecological role in these systems.

The relative abundance of Acidobacteria was low in the Qinghai-Tibetan wetland samples compared with some other wetlands. For example, Acidobacteria was the dominant phylum in a northern peatland [2] and a bog of the glacial Lake Agassiz peatland [4]. These two sites have pH values of ∼4. In contrast, Acidobacteria was not highly abundant in the fen sediments of the Lake Agassiz peatland [4], which had a slightly higher pore water pH value of ∼6. The average porewater pH of the HY and DX peatland was 6.3 and in MD it was 7.8, which could explain the relatively low Acidobacteria abundances observed. In agreement with our results, a previous study also did not detect Acidobacteria by DGGE in peatland of Zoige plateau close to where HY wetland is located [14].

Methane in natural wetlands is the final product of the anaerobic degradation of organic matter and performed by methanogenic archaea [42]. A large part of methane is oxidized by methanotrophs before it reaches the atmosphere [43] and therefore both methanogens and methanotrophs are the key players in the methane-cycling of natural wetlands. Methanogens in HY wetland were significantly more abundant than the other two sites, which is consistent with the higher SOC content in this wetland. Methanogens were more abundant in the hollows, which is consistent with their strict anaerobic metabolism and the hollows more likely to be anoxic compared with hummocks. Methanobacteria were the most abundant methanogens in HY and DX wetlands, followed by Methanomicrobia. The opposite was found in the MD wetland with higher relative abundance of Methanomicrobia followed by Methanobacteria. Previous reports from the Zoige wetland on the Qinghai-Tibetan plateau identified members of Methanomicrobia (*Methanosarcinales* and *Methanomicrobiales*) as the dominant methanogens [8], [44]. This variability suggests that the methanogen community composition in these wetlands might be a result of multiple factors and further studies should be performed to determine which factors play key roles in structuring methanogen populations. Among methanotrophs, *Methylocystis* was the most abundant, which is consistent with the results of a *pmoA* gene pyrosequencing study of the HY site [10]. The *pmoA* gene encodes a subunit of the methane monooxygenase enzyme and is frequently used to identify methanotrophs in environmental samples. A comparison between these studies showed relatively fewer Type I methanotrophs (Gammaproteobacteria) detected by 16S rRNA than *pmoA* gene pyrosequencing. This can be explained by many of the Type I-associated *pmoA* clades being associated with uncultivated methanotrophs [10], which in many cases would not be identified based on 16S rRNA sequences depending on their phylogenetic distance to known methanotrophs.

Our analysis of the relative abundance of various microbial phyla, classes, orders and especially OTUs, showed that the major microbial community structures were significantly different among wetland locations and microtopographies. The data indicated that pH was one of the major soil properties influencing the microbial community composition and diversity in wetlands of the Qinghai-Tibetan Plateau. This effect of environmental pH has also been found in other terrestrial and aquatic ecosystems [16], [17], [45]. The influence of pH on microbial community composition is probably due to the narrow pH ranges for optimal growth of bacteria [16]. Among the soil properties, SOC, AP, TN, and SM were also important contributors to the variations in the microbial community within these wetland soils. All of these variables are related to nutrient availability, which will have obvious implications for microbial and plant growth. A total of 64% of community variance could be explained by these environmental factors, leaving 36% unexplained. Therefore, additional factors such as plant species [46] and some unmonitored variables are likely to have further contributed to the differences in microbial community structure.

## Conclusions

In summary, microbial communities of three Sedge-dominated natural wetlands in the Qinghai-Tibetan Plateau were investigated by 454-pyrosequencing in this study. The differences of soil microbial community structure between the wetlands at different geographic locations and microtopographies could be clearly distinguished by pyrosequencing. The CCA results suggested that there was a clear association between microbial community structure and wetland soil properties. These results provide insight into the microbial community structure in this distinct ecosystem and identified the main factors shaping microbial community structure, which will lead to a more comprehensive understanding of microbial distribution in this region and wetlands in general.

## Supporting Information

Figure S1 **Map showing the locations of three wetlands sampled on the Qinghai-Tibetan Plateau.**
(TIF)

Figure S2 **Relative sequence abundance of classes Alphaproteobacteria (A), Betaproteobacteria (B), Deltaproteobacteria (C), and Gammaproteobacteria (D) taxonomic assignments among samples from different locations and microtopographies.** Major orders detected with average relative sequence abundance >0.1% are displayed. Column “Others” indicates the combined relative sequence abundance of all the rare orders and of the taxonomically unclassified sequences in this class. Error bars indicate the standard deviation of relative sequence abundance between three replicates.
(TIF)

Figure S3 **Relative sequence abundance of taxonomic assignments to rare bacterial phyla from samples of different wetlands and microtopographies.** Phyla detected with average relative sequence abundance ≤1% between all samples were defined as rare. Only rare phyla with average relative sequence abundance >0.1% are displayed. Column “Others” indicates the combined relative sequence abundance of all the rare phyla with average relative sequence abundance ≤0.1%, candidate divisions and of the taxonomically unclassified sequences. The error bars indicate the standard deviation of relative sequence abundance between three replications.
(TIF)

Figure S4 **Relative sequence abundances of phyla Acidobacteria (A), Bacteroidetes (B), Chloroflexi (C), and Verrucomicrobia (D) in samples from different locations and microtopographies.** Only the classes with average relative sequence abundances >0.1% are displayed. The error bars indicate the standard deviation of relative sequence abundances between three replications.
(TIF)

File S1 Contains Tables S1–S4.
(DOCX)
