# Supplementary material for: Microbial Diversity in Hummock and Hollow Soils of Three Wetlands on the Qinghai-Tibetan Plateau Revealed by 16S rRNA Pyrosequencing
Source: PLoS One. 2014 Jul 31;9(7):e103115. doi: 10.1371/journal.pone.0103115 (PMC4117511; doi:10.1371/journal.pone.0103115)
Supplement: File S1 — Contains Tables S1–S4. (DOCX) [file pone.0103115.s005.docx]

Table S1. Diversity indices of microbial 16S rRNA gene sequences (mean±SD, n=3). Operational taxonomic units (OTUs) were assigned at 3% genetic distance levels. P-values for two-way ANOVA of diversity indices using geographical locations and microtopographies as main factors are presented in the bottom of the table.

| Group | Nseqs | OTUs | Chao1 | Shannon | Coverage |
| --- | --- | --- | --- | --- | --- |
| DXa | 4130 | 1323±137 | 2380±258 | 6.52±0.21 | 0.83±0.02 |
| DXb | 4130 | 875±16 | 1563±65 | 5.74±0.07 | 0.89±0.00 |
| HYa | 4130 | 1194±54 | 2270±69 | 6.15±0.17 | 0.84±0.01 |
| HYb | 4130 | 1314±90 | 2501±157 | 6.35±0.14 | 0.82±0.01 |
| MDa | 4130 | 1191±153 | 2095±351 | 6.08±0.55 | 0.85±0.02 |
| MDb | 4130 | 1174±56 | 2128±76 | 6.11±0.19 | 0.85±0.01 |
|  |  | P-value | P-value | P-value | P-value |
| Location |  | 0.053 | 0.010 | 0.580 | 0.005 |
| Topography |  | 0.028 | 0.068 | 0.184 | 0.031 |
| Location×Topography |  | 0.001 | 0.001 | 0.019 | <0.001 |

Table S2. Results of two-way ANOVA for relative sequence abundances of major phyla and classes in the Proteobacteria phylum using geographical locations and microtopographies as main factors.

| Taxa | Location | Topography | Location×Topography |
| --- | --- | --- | --- |
|  | P-value | P-value | P-value |
| Alphaproteobacteria | <0.001 | 0.004 | 0.065 |
| Betaproteobacteria | 0.743 | 0.115 | 0.642 |
| Deltaproteobacteria | 0.754 | 0.083 | 0.073 |
| Gammaproteobacteria | 0.004 | 0.470 | 0.929 |
| Actinobacteria | 0.053 | 0.033 | 0.892 |
| Bacteroidetes | 0.049 | 0.492 | 0.006 |
| Chloroflexi | 0.108 | 0.005 | 0.001 |
| Acidobacteria | 0.004 | 0.710 | 0.177 |
| Verrucomicrobia | 0.003 | 0.064 | 0.131 |
| Firmicutes | 0.040 | 0.299 | 0.023 |
| Planctomycetes | 0.825 | 0.004 | 0.084 |
| Bacteria-others | <0.001 | 0.009 | 0.133 |

Table S3. Results of two-way ANOVA for relative sequence abundances of major classes in the phyla Acidobacteria, Bacteroidetes, Chloroflexi and Verrucomicrobia using geographical locations and microtopographies as main factors.

| Phylum | Class | Location | Topography | Location×Topography |
| --- | --- | --- | --- | --- |
|  |  | P-value | P-value | P-value |
| Acidobacteria | Acidobacteria | 0.001 | 0.574 | 0.054 |
|  | Holophagae | 0.287 | 0.067 | 0.428 |
| Bacteroidetes | Sphingobacteria | 0.956 | 0.004 | 0.320 |
|  | Bacteroidia | 0.142 | 0.643 | 0.011 |
| Chloroflexi | KD4-96 | 0.032 | 0.214 | 0.007 |
|  | Anaerolineae | 0.001 | <0.001 | 0.076 |
| Verrucomicrobia | Spartobacteria | 0.658 | <0.001 | 0.011 |
|  | OPB35 | 0.001 | 0.002 | 0.005 |

Table S4. Partitions and Mantel analysis of environmental factors on the structure of bacterial communities.

| Factor | Explained (%) | Mantel statistic r | Significance |
| --- | --- | --- | --- |
| TN | 11.02 | 0.362 | 0.003 |
| SOC | 11.19 | 0.425 | 0.002 |
| AP | 10.17 | 0.522 | 0.001 |
| pH | 13.69 | 0.735 | 0.001 |
| C:N | 9.35 | 0.253 | 0.008 |
| SM | 11.46 | 0.493 | 0.001 |
| PB | 8.04 | 0.081 | 0.151 |
| T | 12.85 | 0.567 | 0.001 |
| P | 12.23 | 0.606 | 0.001 |
| A | 10.76 | 0.356 | 0.003 |
